# Connecting groups and behaviours: A network analysis of identity‐infused behaviours

**DOI:** 10.1111/bjso.12674

**Published:** 2023-08-02

**Authors:** Emily A. Hughes, Samuel Ellis, Joanne R. Smith

**Affiliations:** ^1^ University of Exeter Exeter UK

**Keywords:** group membership, network analysis, social identity

## Abstract

Research in the social identity tradition acknowledges the multiplicity of our identities and the implications that identity compatibility has for our health and well‐being. However, current measures of multiple group membership have not yet captured the richness and complexity of our social identity networks at the wider sample level, and data regarding the different behaviours typically associated with different group memberships are scarce. Adopting a network approach, we explore the co‐occurrence of different group memberships within an individual (identity‐by‐identity network), the behaviours that are shared among identities (behaviour‐by‐identity network), and whether identities that are shared also share common behaviours (identity‐by‐behaviour network). An online survey asked participants (*N* = 286) to list the groups they are part of, as well as the behaviours viewed to be typical of group members. The networks identified several identities and behaviours to significantly co‐occur at a rate both higher and lower than chance. Networks were found to be low in modularity; there was no evidence of clustering within the data. Permutation analyses demonstrated the overall structure of the networks to be significantly different than expected by chance. The co‐occurrences identified serve as a meaningful resource for those conducting research into identities, group norms and their associated behaviours.

## BACKGROUND

Many of the behaviours that we perform in everyday life are identity infused, meaning that they are driven by what it means to be part of a specific social group. Individuals also possess multiple social identities, which are typically associated with distinctive behavioural profiles at the intergroup level. For example, an individual may identify as both a student and as a member of their sports team. However, while both these groups may engage in drinking behaviour, members of a sports team may also engage in healthier behaviours, such as exercising, to an extent that students do not. Similarly, studying may be a behaviour typical of the student identity, but one that is not associated with being a member of a sports team. The content of our social identities guides our behaviour depending on the identity that is salient at any given time. However, data regarding the co‐existence of social identities within an individual are scarce, as is our knowledge of the behaviours associated with particular identities.

Past research has developed procedures to map different social identities at an individual level (i.e. where the identity‐by‐identity network relevant to each individual is mapped independently; Cruwys et al., 2016). However, this approach does not tell us anything about the relationships among social identities at a wider sample level. Thus, numerous questions are left unanswered. For example, which identities are found, statistically, to co‐occur significantly more than others across individuals? How are different identities structurally organized within the population? Do identities form meaningful sub‐groups (i.e. modular clusters), or is there a high level of interconnectivity among identities – meaning that individuals identify in a complex and diverse manner, and there are no ‘sets’ of commonly held identities?

Population‐level network approaches (i.e. where identity‐by‐identity data are aggregated and mapped across individuals) provide researchers with the methodological and analytical tools to address these questions. Broadly speaking, these approaches allow us to quantify, statistically, the structural relationships – and the strength of ties – among concepts. Through data visualization, we can grasp the ‘bigger picture’ of relations across individuals, allowing us to draw stronger conclusions regarding their generalizability. Network analytic methods have been used in the social identity literature to measure links among norms (Paluck et al., 2016; Paluck & Shepherd, 2012), attitudes and behaviours (Dinkelberg et al., 2021; Maher et al., 2020; Paluck, 2011) – but provide a novel analytic approach to the modelling of social identities. When applied to the study of relations among identities, the approach facilitates a broader, more concrete understanding of the multiplicity of group memberships – and the groups that are found to frequently co‐occur – than can be attained through existing methods.

Over and above providing a proof of concept – that network analytic methods can be applied to model and quantify the co‐occurrence of different group memberships within individuals at the wider sample level – the insights from this research are important methodologically, theoretically and practically. Methodologically, the research makes an important contribution by providing researchers with a clear way of visually mapping, quantifying and disentangling several sub‐dimensions of identity across individuals – including both group membership, and the content of this group membership (e.g. normative behaviour). Theoretically, the approach establishes the structure and complexity of intrapersonal identification and group‐behaviour associations at a population level, which is presently unknown. Practically, this research has the potential to inform and improve the efficacy of identity‐based behaviour change interventions – which typically target behaviour that is normative for a given group (LaBrie et al., 2013), without appreciating that this same behaviour may also be associated with, and therefore reinforced by, other identities.

### Personal versus social identity

Both social identity theory (SIT; Tajfel, 1978; Tajfel & Turner, 1979, 1986) and self‐categorization theory (SCT; Turner et al., 1987) distinguish personal and social identity, such that human interaction is seen to range on a spectrum from being purely interpersonal to purely intergroup. From a social identity perspective, a shift from one endpoint of this spectrum to the other results in a shift in how individuals see both themselves and others; this distinction influences the cognitions, attitudes and behaviours that comprise an individual's self‐concept (Tajfel & Wilkes, 1963). SCT retains, but expands upon, the core of this principle – suggesting that identity can be understood at varying degrees of abstraction and inclusivity. SCT proposes there to be three levels of self‐categorization important to the self‐concept: “human identity”, “social identity” and “personal identity” (Hornsey, 2008, p. 208).

SCT focuses primarily on predicting contextually dependent categorization with a single group membership (Turner, 1982; Turner et al., 1987). The theory proposes that for a specific social identity to become salient in any given social situation, not only must the differences between groups be greater than the differences within them (i.e. the meta‐contrast principle), but the objective between‐group differences must conform to normative beliefs surrounding what it means to be a group member (i.e. the normative fit principle; Turner et al., 1987). Importantly, the category must also be cognitively accessible in the moment. SCT uncovered the cognitive processes involved in self‐categorization with a single social identity and brought attention to the importance of both accessibility and fit (Oakes, 1987; Oakes et al., 1991).

### Social complexity and multiple identities

Building on the social identity approach, additional accounts have been proposed to explain the complexities surrounding social identification processes and to illustrate how individuals can cognitively reconcile their multiple different social identities within the self‐concept. These models emphasize the importance of maintaining social identities as distinct entities while allowing linkages to be formed among them. For example, the Social Identity Complexity Model (Roccas & Brewer, 2002) details a continuum of four different classes of cognitive representation – moving from reducing multiple divergent social identities to a single social category, or adopting only one primary group identity, to simultaneously recognizing and reconciling non‐convergent social identities in their most inclusive form. Similarly, the Cognitive‐Developmental Model of Social Identity Integration (CDMSII; Amiot & de la Sablonnière, 2010; Amiot et al., 2007) accounts for both the multiplicity of social identities and their assimilation within the self‐concept, while also specifying how these cognitive configurations change over time. Ultimately, such models acknowledge that individuals organize their social identities in a more, as opposed to less, complex manner – such that the multiple group memberships can contribute to the self‐concept both positively and distinctively.

### The benefit of multiple identities

Recent research has investigated the psychological consequences of multiple group membership. Considerable evidence has been found for the association between multiplicity of group memberships and enhanced well‐being – particularly in the face of illnesses and injuries such as stroke (Haslam et al., 2008), dementia (Jetten et al., 2010) and brain injury (Jones et al., 2011). Being a member of multiple groups is also associated with increased access to resources during transitional life periods: the more group memberships an individual has, the more likely they will be to adopt and identify with a new group (i.e. identifying as a student throughout the transition to university; Iyer et al., 2009), and be buffered against the potential negative consequences of identity transitions (Iyer et al., 2009; Thotis, 1983). Research also finds that individuals are better able to respond to novel and aversive physical challenges when more group memberships are cognitively salient (Jones & Jetten, 2011).

### The importance of identity compatibility

Although early accounts of social identity complexity adopted a ‘more the merrier’ approach to the outcomes of multiple group memberships (Roccas & Brewer, 2002), later models, such as the CDMSII (Amiot et al., 2007), have suggested that belonging to multiple social groups will be more beneficial when integrated and reconciled, versus when they are in conflict. This represented a shift in focus from identity *complexity* to identity *compatibility* (Ramarajan, 2014; Rosenthal et al., 2011). Indeed, there is a positive correlation between identity quantity (i.e. multiple group memberships) and well‐being, but only if these identities are perceived to be both important and compatible with one another (Brook et al., 2008; Iyer et al., 2009). When identities are perceived to be important, but incompatible, then more group memberships are associated with reduced well‐being. The compatibility of group memberships appears to moderate the ‘more the merrier’ effect.

### Measurement of multiple identities

A variety of measures have been designed to index multiple group memberships. These include open‐ended measures of identity quantity (Brook et al., 2008), as well as scale measures of relations among pairs of identities (e.g. conflict; Benet‐Martínez & Haritatos, 2005; Settles, 2004) and the overall structuring (e.g. hierarchy and relative identification; Stryker & Serpe, 1982) of group memberships (see Ramarajan, 2014 for a review). These measures focus on measuring the multiplicity of an individual's social group network in a general sense and are relatively easy to administer and score. More recent measurement tools have aimed to provide richer, more comprehensive data on a person's social group networks. For example, Social Identity Mapping (SIM; Bentley et al., 2020; Cruwys et al., 2016) has been used to explore – simultaneously – the number of group memberships an individual has, the importance of each social group, the similarity among these groups and their compatibility. Individuals are asked to write down the name of each group they belong to on different‐sized Post‐it notes – the larger the Post‐it note, the more important the group. Participants are also asked to spatially cluster similar groups near one and other, and dissimilar groups far apart, on a page. Compatible groups are to be joined by straight lines, and incompatible groups by jagged lines. Procedures such as SIM adopt a bottom‐up, idiographic approach (i.e. one in which participants qualitatively list their own social identities and draw their own conclusions regarding their interrelations). In doing so, they bridge the gap between the complexity with which social psychological constructs are communicated theoretically (e.g. multiple group membership and group compatibility within the social identity approach) and the ways in which these constructs are typically measured (e.g. via Likert scale).

### Benefits of a network approach

In recent years, there has been a call for the adoption of a network analytic approach in the measurement of social identities – moving beyond capturing identities acting independently, and instead acknowledging and understanding multiple, fluid relationships among many identities (Ramarajan, 2014). While the development of techniques such as SIM brings us a step closer to understanding the richness of social identity networks, SIM focuses on conceptualizing and mapping multiple identities at the level of the *individual*. This is helpful in applied therapeutic settings (Cruwys et al., 2022; Haslam et al., 2016, 2019), but cannot tell us about the co‐occurrences of multiple social identities at a broader population level. Moreover, it cannot tell us anything statistically about the identities that are found to co‐occur significantly more often than others. Conversely, a population‐level network approach applies statistical analyses to theoretically informed network models – allowing stronger conclusions to be drawn regarding the broader overall structuring of a network, and the strength of ties among concepts within it.

Network models have allowed researchers to examine (Paluck & Shepherd, 2012), and intervene in (Paluck et al., 2016), the transmission of social norms and harassment behaviour at school level. Recently, network models have helped to confirm network‐related hypotheses about the relationships among beliefs in different conspiracy theories – providing statistical support for the theoretical assumption that beliefs support one another in a mutually reinforcing network (Williams et al., 2022). Networks are also particularly useful in identifying polarization and clustering in data sets; they have been used to map attitudes – linked by individuals who share them – as a novel means of detecting attitudinal alignment and the emergence of polarized opinion‐based groups that go on to demonstrate differences in their behaviour (Dinkelberg et al., 2021; Maher et al., 2020). Similarly, geographically based clusters of prosociality have also been identified in networks of charitable giving (Chapman et al., 2022). In numerous domains, the method offers a straightforward and theoretically informed way of conceptualizing, inductively identifying and quantifying connections among constructs in a network.

### The present research

The co‐occurrence of different group memberships has yet to be investigated statistically to explore the multiple identities that occur – and co‐occur – more often than others. To the best of our knowledge, the present research is the first to adopt a network approach to both collecting and analysing these data at a wider sample level. By asking individuals to list the multiple groups to which they belong – and creating identity‐by‐identity networks – we explore the interrelations among these social identities and uncover which identities co‐occur at a rate that is both higher, and lower, than expected by chance. This analytic approach allows us to clearly map and visualize these co‐occurrences – acknowledging the density and richness of these identity networks in a way that traditional self‐report measures cannot. The approach also helps us better understand the structure among many relationships that form a greater network and avoids imposing pre‐determined social categories upon participants.

Our approach might also provide insight into why certain identities may be less compatible than others by exploring the compatibility of their associated behaviours. Research into role conflict (see Burke, 2006) documents the negative consequences experienced by those occupying conflicting roles, such as students who compete in athletics (Settles et al., 2002). However, this research attributes the incompatibility between roles to the different demands of a particular group or individual more generally, rather than focusing specifically on behavioural^1^ incompatibility among identities. For example, because drinking behaviour is associated with the student identity (Zhou & Heim, 2016), you would assume this identity to be more compatible – and co‐occur more frequently – with other identities that are also associated with drinking (e.g. sports group identity; Zhou & Heim, 2016) than those that are typically not (e.g. organizational identity). Hence, the network approach of the present research identifies which behaviours are shared by identities (behaviour‐by‐identity network) and put differently, whether identities that are shared also share common behaviours (identity‐by‐behaviour network). In doing so, these data provide a unique resource for those wishing to identify specific groups and behaviours to feature in future research.

As previous research has not established the behaviours associated with particular groups, insights from the present research may be particularly useful for those looking to target particular groups for intervention in order to modify maladaptive behaviours that are highly identity infused. Measuring, and mapping, the normative perceptions of behaviour via network analytic methods is particularly beneficial in terms of informing the design of interventions – given that norm‐based interventions are commonly used as a means of achieving behaviour change (LaBrie et al., 2013; Lewis & Neighbors, 2006; Neighbors et al., 2004). Specifically, behaviour‐by‐identity and identity‐by‐behaviour networks will enable practitioners to identify behaviours that are normative for multiple identities – or identities that share the performance of normative behaviours – and target the descriptive norms associated with each group. This is likely to be advantageous over targeting maladaptive behaviour in relation to a single group membership – as is typical of behaviour change interventions – given that the behaviour may continue to be reinforced by the other identities associated with its performance.

## METHOD

### Participants and design

The online survey recruited a total of 286 participants through both an undergraduate participant pool and SurveyCircle.^2^ Participants' age ranged from 18 to 58 years (*M*_age_ = 22.29, *SD* = 6.45). The majority of the sample were female (68.5%), students (68.2%) and of White ethnicity (65%). Detailed demographic information can be found in Table 1. For their participation, individuals were granted either 0.5 course credits or three survey reward points. Ethical approval was obtained prior to data collection.

**TABLE 1:** Detailed demographic information.

	*N* = 286	
	*n*	%
Age		
18–30	214	74.6
31–40	10	3.3
41–50	4	1.2
51+	3	0.9
Gender		
Male	43	15.0
Female	196	68.5
Transgender	1	0.3
Other	3	1.0
Prefer not to say	1	0.3
Ethnicity		
White	186	65.0
Black or African American	0	0
American Indian or Alaska Native	0	0
Asian	46	16.1
Native Hawaiian or Pacific Islander	0	0
Other	7	2.4
Prefer not to say	3	1.0
Employment		
Full‐time employed	24	8.4
Part‐time employed	10	3.5
Self‐employed	2	0.7
Unemployed	3	1.0
Student	195	68.2
Retired	0	0
Unable to work	0	0
Other	5	1.7
Prefer not to say	1	0.3

*Note*: Values may not sum to 100% due to missing demographic data.

### Procedure

After providing informed consent, participants completed the online questionnaire, which assessed the different group memberships/social identities possessed by each participant, behaviours typically performed by each group and social demographics.^3^ Table 2 outlines descriptive statistics regarding the number of social identities reported per person, the number of behaviours reported per identity and the number of behaviours reported per person.

**TABLE 2:** Descriptive statistics for number of social identities and behaviours reported.

	*M*	*SD*	Mode	Range
Social identities	5.20	1.95	5	1–10
Behaviours (per identity)	2.06	1.26	1	1–10
Behaviours (per person)	8.65	5.79	6	1–33

*Note*: The number of social identities reported was limited to 10 per participant. The number of behaviours reported alongside each identity was not limited.

#### Group membership

Participants were first presented with 10 free‐entry text boxes – labelled “Group 1” through “Group 10” – and were asked to write down as many different group memberships/identities as possible. Participants were instructed to write each group in a separate box and to only write down groups to which they belong. This list of identities was carried forward to the next page of the survey, where it re‐appeared on screen.

#### Behaviour

To assess the behaviours typically associated with different group memberships, a free‐entry text box was located next to the name of each group that a participant had previously mentioned. Here, participants were required to list the different behaviours that members of each group typically perform. Each different behaviour was to be separated by a comma, and individuals were encouraged to provide as many examples as possible for each identity.

#### Demographics

Participants were asked to indicate their age, gender, ethnicity and employment status. At the end of the study, participants were debriefed as to the nature of the research and provided with the relevant contact details to address any queries. All materials, data and code are available on the project Open Science Framework (OSF) page.

### Network analysis

#### Creating the networks

Adopting network methods, three separate networks were devised. To address our first research question regarding the identities found to co‐exist across the population, an identity‐by‐identity network was created – whereby the nodes represent individual identities, and the edge weight represents the normalized number of people the pair of identities are found in. To address our second research question regarding the behaviours associated with identities, two networks were created to present the identity–behaviour relationships differently; a behaviour‐by‐identity network to capture the behaviours that are shared among identities, and an identity‐by‐behaviour network to capture the identities that share behaviours. In the behaviour‐by‐identity network, the nodes represent behaviours, and the edge weight represents the number of identities sharing the behaviour. In the identity‐by‐behaviour network, the nodes represent identities, and the edge weight represents the number of behaviours shared among linked identities.

For each pair of nodes in each network, we calculated the edge weight as the number of times that both nodes were reported by the same participants (*x*), as a proportion of the total number of times that the two nodes were reported in the data either together (*x*) or apart (*y*_*a*_, *y*_*b*_). More formally, the edge weight between a pair of nodes – *A* and *B* – was calculated as:

$$\frac{x}{x+y_{a}+y_{b}}$$

This methodology is identical to the Simple Ratio Index commonly used in the study of animal social behaviour (Whitehead, 2008). The advantage of this index is that it offers some control for the number of times that particular nodes in the network are observed overall.

Two versions were created for each of the three network types; a full unfiltered network and a filtered network (see Table 3). The unfiltered networks featured all valid^4^ identities and behaviours mentioned by participants (see Appendix B). As many identities and behaviours were only identified by one, or a small number of, participants – and therefore obscured more general patterns at the population level – identities and behaviours mentioned most frequently were extracted. Hence, the filtered networks featured only nodes observed more than three times within the data set. All analyses were conducted on the filtered networks.^5^

**TABLE 3:** Number of nodes in unfiltered and filtered networks.

Network type	Network version	N nodes
Identity‐by‐Identity	Unfiltered	138
	Filtered	62
Behaviour‐by‐Identity	Unfiltered	83
	Filtered	36
Identity‐by‐Behaviour	Unfiltered	86
	Filtered	49

To explore the broader generalizability of our observed network structure (i.e. in terms of density and modularity) across individuals and determine whether this structure may depend on the categories used in our original data cleaning procedure (see Appendix A), identities were re‐coded to reflect the overarching categories present within the data (e.g. national identity, gender identity, political identity, etc.), and data were reanalysed (see Appendix C for the re‐coding procedure, and Appendix D for the filtered^6^ network figures and summary statistics resulting from re‐analysis).

#### Network structure

We focused on two features of the filtered networks: (1) the overall structure of the network, and (2) which nodes share stronger, or weaker, edges than expected by chance.

The first analysis aimed to characterize the structure of the network as a whole. We used three metrics to understand the structure of our networks: density, modularity and non‐random structure. Density is the number of edges in the network as a proportion of the total possible number of edges in the network. It was calculated as:

$$\text{Density}=\frac{2E}{n\left(n-1\right)}$$

where *E* and *n* are the number of edges and nodes, respectively, in the network (see Croft et al., 2008). A density of 1, therefore, represents a network where all nodes are connected to all other nodes (100% of all possible edges are present in the network), whereas a density of 0.1 means that 10% of all possible edges are present in the network. This measure allowed us to describe the extent to which each network is integrated and interconnected.

Modularity characterizes the extent to which a network can be separated into different subgroups (known as modules). More formally, modularity describes the proportion of edges occurring within, rather than between, subgroups. A network separated into distinct subgroups with no connections between them would, therefore, have a modularity of 1, and a network with no separation into subgroups would have a modularity approaching 0. Various algorithms have been devised to identify the sub‐groups within the network. We used the walktrap algorithm, which uses repeated four‐step random walks between nodes over the network – weighted by edge weight – to identify subgroups (Pons & Latapy, 2006), and then used these subgroups to calculate modularity. Other algorithms gave qualitatively similar outputs.

We quantified the centrality of nodes in the networks using betweenness centrality. Betweenness centrality assesses the importance of a node in connecting different areas or substructures of the network (Whitehead, 2008). This measure is calculated by taking the shortest paths (weighted by edge strength) between all pairs of nodes in the network. The betweenness centrality of a given node is the number of shortest paths passing through that node (Newman, 2004).

We also investigated the correlation between the identity‐by‐identity network and the identity‐by‐behaviour network using a Mantel test (Mantel, 1967). Mantel tests are matrix correlation tests, and can be interpreted in the same way as an ‘ordinary’ correlation analysis: where *r* = 0 means no correlation, *r* = 1 a complete positive correlation and *r* = −1 a complete negative correlation. Networks can be considered as an *N* x *N* association matrices *A*, where *N* is the number of nodes and *A*_*i*,*j*_ = the weight of the edge between nodes *i* and *j*. Only nodes present in both networks were included in the association matrices (*n* = 44).

Finally, we calculated whether the structure of the network differed from that expected if edges were formed at random. To answer this question, we permuted the observed data to create randomized data sets based on the null hypothesis that the structure of the networks is random. We then compared the network structure of these permutated data sets to the structure of the random data sets; if the observed data set differs from the permutated data sets, it suggests that the network is not organized at random. We used the Bejder permutation method (sometimes called datastream permutations), which creates randomized data sets by swapping node‐label pairs between samples (Bejder et al., 1998). For the identity‐by‐identity and identity‐by‐behaviour networks, the ‘node‐labels’ are the identities reported by study participants. For the behaviour‐by‐identity network, the ‘node‐labels’ are the reported behaviours. The samples are the sets of responses connected to a particular participant. That is, all the identities reported by a single individual for the identity‐by‐identity network, and the behaviours linked to a given identity by a participant in the identity‐by‐behaviour and behaviour‐by‐identity networks. This permutation method holds constant (1) the number of participants, (2) the number of responses each participant gives and (3) the number of times each response (behaviour or identity) is returned in the data – while varying which responses each participant gives. We performed 10,000 permutations, and each permutation therefore represented a ‘random world’ – where there are no associations between identities and behaviours, but the structure of the data is the same as observed in the real responses. For each permuted network, we then calculated the coefficient of variation of edge weights as a metric of network structure. We compared the distribution of these permutated coefficient of variations to the observed coefficient of variation. The proportion of permuted values that are larger than the observed value is, therefore, the probability that the network structure is generated by random processes (proportions calculated as Ruxton & Neuhäuser, 2013). This methodological pathway was developed and is widely used to study animal social networks, where there is also often uneven sampling between individuals and groups (see Brask et al., 2021; Croft et al., 2008; Hobson et al., 2021; Whitehead, 2008), but are here applied for the first time to understand the structure of social identity and behaviour networks.

#### Preferred and avoided associations

As well as characterizing the structure of the networks as a whole, we also examined which nodes in the network have stronger or weaker edges connecting them than expected by chance.^7^ In the identity‐by‐identity networks, for example, this analysis identified which pairs of identities are reported by the same participant more or less often than expected by chance. In the identity‐by‐behaviour networks, the analysis identified which identities share more or fewer behaviours than expected by chance. Finally, in the behaviour‐by‐identity networks, the analysis identified which behaviours are shared by more or fewer identities than expected by chance. In the study of social networks – where nodes are individuals and the edges between them are the strength of their social connection – this analysis is used to identify ‘preferred’ and ‘avoided’ social partners, terminology we continue to use here.

Preferred and avoided associations were identified using the same permutation methods used to identify non‐random network structure. In each permutation, we calculated the strength of association between every pair of nodes in the network: the permuted edge strength (Whitehead, 2008). The distribution of these permuted edge weights is the edge strengths we would expect to see if nodes were reported by participants at random. As for the non‐random structure analysis, the proportion of permutated strengths greater than the observed strength is the probability that the pairs are associating more often than expected by chance, and the proportion of permuted strengths less than the observed value is the probability that the nodes are associating less often than expected by chance. We used a two‐tailed test with *α* = .95, and therefore pairs of nodes associating more often than .975 of the permutated values are considered to be preferred associates, and those less often than .025 of the permuted values are considered to be avoided associates.

## RESULTS

All analyses^8^ and plotting of networks were conducted in R v4.1.1 (R Development Core Team, 2021) using the dplyr, asnipe, igraph, ForceAtlas2, aninet, ggraph, tidygraph and cowplot packages (Alvarez, 2015; Csárdi & Nepusz, 2006; Farine, 2013; Pedersen, 2021, 2022; Weiss, 2020; Wickham et al., 2019; Wilke, 2020).

### Identity‐by‐identity network

**FIGURE 1:**
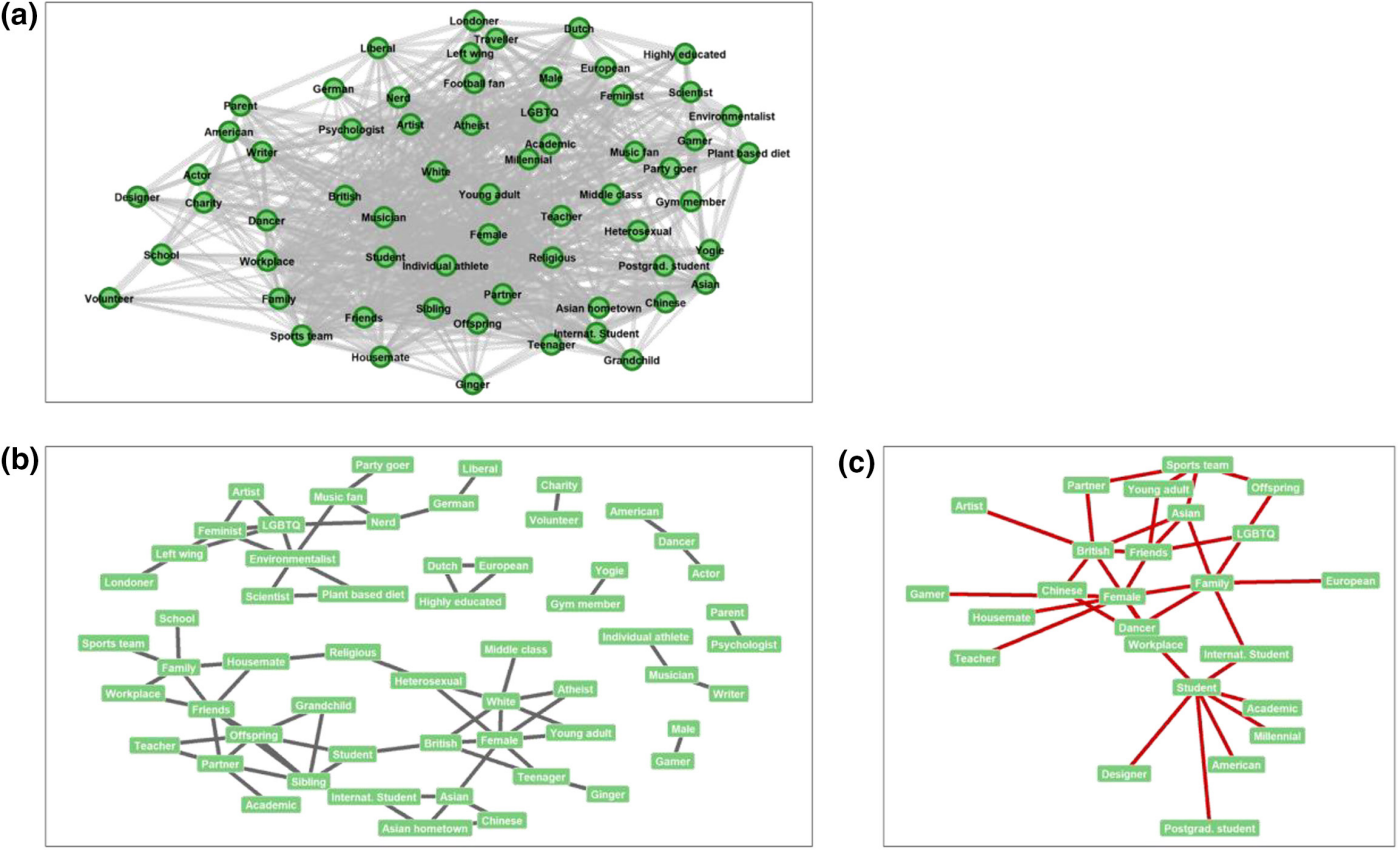
Identity‐by‐identity networks.

The filtered network (see Figure 1a) demonstrated low modularity (.22; unfiltered = .43) – suggesting that there is little clustering within the data,^9^ and that connections between nodes within modules are no denser than connections between nodes in different modules. The overall, relatively high, density (.44) of the filtered network (i.e. the proportion of possible connections present in the network) further suggests a relatively low degree of substructure within the network. Identities with the highest betweenness centrality (see Table 4) include ‘female’ and ‘student’ – falling on the shortest path between other nodes 153 and 147 times respectively. In other words, these identities are often situated in‐between others, suggesting that they often serve as bridges from one part of the network to another, and are therefore important in determining network structure. Permutation analyses also demonstrated the overall structure of the network to be significantly different than expected by chance (*p* = .004).

**TABLE 4:** Identities with the 10 highest betweenness centrality scores.

Ranking	Identity	Betweenness
1	Female	153
2	Student	147
3	Asian	130
4	Scientist	120
5	Offspring	97
6	White	94.5
7	American	84.5
8	Partner	79
9	Nerd	77
10	European	73
10	LGBTQ	73

*Note*: Betweenness centrality scores for all 62 identity nodes can be found under the data and analysis section of the project OSF page.

Figure 1b depicts the 57 pairs of identities found significantly to occur in the same person more often than expected by chance. Triads of significantly co‐occurring identities can be seen to form around identities relating to sexual orientation and activism (e.g. LGBTQ‐feminist‐environmentalist, LGBTQ‐feminist‐artist, LGBTQ‐feminist‐left wing), nationality (e.g. Asian‐Asian hometown‐international student, Asian‐Asian hometown‐Chinese), gender and ethnicity (White‐female‐young adult, White‐female‐heterosexual, White‐female‐British, White‐female‐atheist), family and friends (family‐friends‐workplace, family‐friends‐housemate) and identifying as a son or daughter (offspring‐friends‐partner, offspring‐partner‐sibling, offspring‐sibling‐grandchild, offspring‐sibling‐student, offspring‐teacher‐partner). There are also several pairs of identities that are significantly associated with one another but do not share this interconnectivity with other identities (e.g. charity‐volunteer, male‐gamer, gym member‐yogie, parent‐psychologist). Figure 1c depicts the 25 pairs found significantly to occur in the same person less often than expected by chance – the majority of these were also stand‐alone pairs of associations.

### Behaviour‐by‐identity network

**FIGURE 2:**
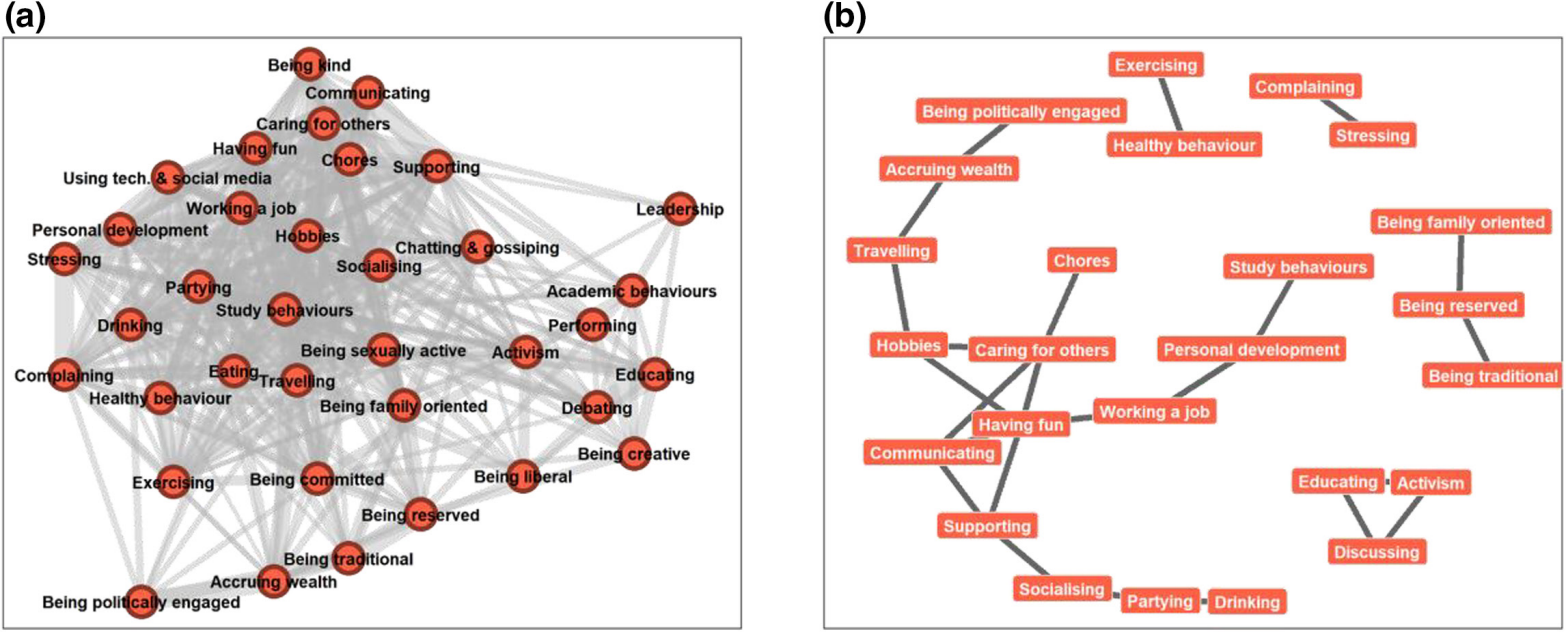
Behaviour‐by‐identity networks.

The filtered network (see Figure 2a) demonstrated low modularity (.14; unfiltered = .37) – again suggesting that there is little clustering within the data and that connections between nodes within modules are no denser than connections between nodes in different modules. The overall density of the filtered network (.56) further demonstrates there to be a relatively high number of connections, with a lack of substructure within the network. Permutation analyses also demonstrated the overall structure of the network to be significantly different than expected by chance (*p* < .001).

Figure 2b depicts the 25 pairs of behaviours significantly associated with the same identities more often than expected by chance. Behaviours shared among identities include health‐related behaviour (e.g. healthy behaviour exercising), education and social change‐focused behaviour (e.g. educating‐activism‐discussing), occupational behaviour (e.g. personal development‐working a job, personal development‐study behaviours), caregiving behaviour (e.g. communicating‐caring for others, communicating‐supporting) and social activities (e.g. partying‐drinking, partying‐socializing). Regarding the pairs of behaviours significantly associated with the same identities less often than expected by chance, there were only two; the behaviour of ‘caring for others’ paired with ‘alcohol consumption’ (*p* = .024) and the behaviour of ‘socializing’ paired with ‘discussing’ (debating; *p* = .021).

### Identity‐by‐behaviour network

**FIGURE 3:**
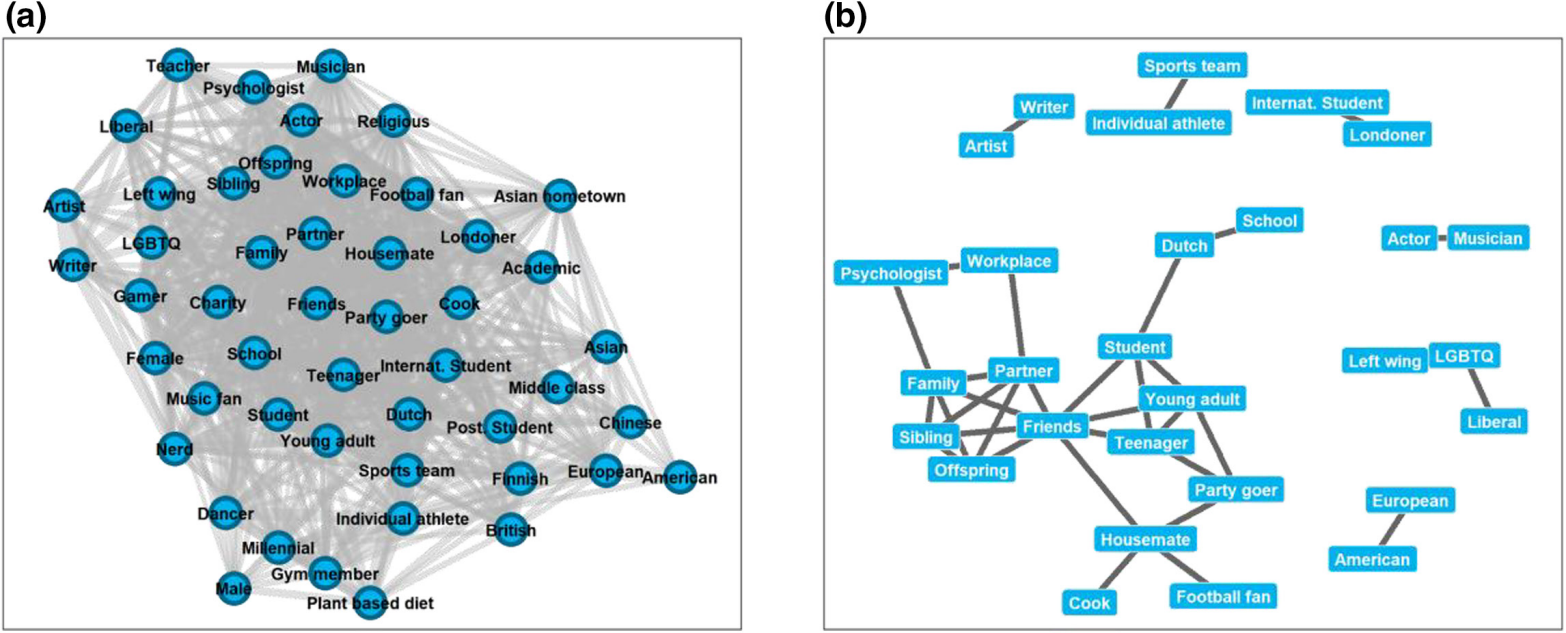
Identity‐by‐behaviour networks.

The filtered network (see Figure 3a) demonstrated low modularity (.15; unfiltered = .21) – again suggesting little to no clustering within the data. The overall density of the filtered network (.59) further demonstrates there to be a relatively high number of connections, with a lack of substructure within the network. Permutation analyses demonstrated the overall structure of the network to be significantly different than expected by chance (*p* < .001). Correlational analyses found no significant association (Mantel test: *r* = .04, *n* = 44, *p* = .33) between the structure of identity‐by‐identity and identity‐by‐behaviour networks, suggesting that the likelihood of an individual sharing any two identities is not predicted in whether those two identities share a behaviour (and vice versa).

Figure 3b depicts the 29 pairs of identities significantly associated with the same behaviours more often than expected by chance. Identities sharing behaviours include sporting identities (e.g. sports team‐individual athlete), sexual and political orientation identities (e.g. LGBTQ‐left wing, LGBTQ‐liberal), artistic identities (e.g. artist‐writer, actor‐musician), occupational identities (e.g. psychologist‐workplace), familial identities (e.g. partner‐family‐sibling‐offspring‐friends) and age group related identities (e.g. young adult‐teenager‐party goer, young adult‐teenager‐friends‐student); none of the identity pairs were found to be significantly associated with the same behaviours less often than expected by chance.

## DISCUSSION

The present research applied a network approach to investigate the co‐occurrence of different group memberships within individuals, the behaviours that are shared among these group memberships, and whether identities that are shared also share common behaviours. Across all networks, results demonstrated the network structures to be significantly different than expected by chance, highlighting the co‐occurrences among identities and behaviours to be meaningful. Identity‐by‐identity networks demonstrated that a large number of identities frequently co‐exist within the same individual. Interconnectivity was demonstrated among identities – predominantly along the lines of nationality and culture, family, physical activity, education and activism. Identities were also connected via shared behaviours along similar lines; organizational, familial, sporting, artistic, age and activism‐based identities tended to have similar behavioural profiles. However, the networks revealed a low degree of modularity, and a relatively high network density – meaning that while each network possessed a relatively high number of connections between nodes, connections within modules were no denser than connections between modules. In other words, these connections did not form statistically notable clusters or subgroupings.

Network analytic methods have been used to map relations among social psychological constructs such as group norms (Paluck et al., 2016; Paluck & Shepherd, 2012) and attitudes (Dinkelberg et al., 2021; Maher et al., 2020) in relation to behaviour, but the current research is, to our knowledge, the first to apply these methods to the study of group memberships and their associated behaviours. The research clearly identifies a host of identities and behaviours found to significantly co‐occur at rates both higher, and lower, than chance – demonstrating the utility of a network approach to both the measurement and analysis of these social psychological constructs. It, therefore, serves as a unique and important resource (i.e. in terms of the methodological and statistical approach) for those wishing to apply these techniques in their own research – particularly given the open access to materials and associated code. Moreover, given the flexibility of the approach, it has the potential to be adapted to map several indices of the relations between social identities (e.g. importance, similarity, positivity, compatibility), and disentangle many of the sub‐dimensions of identity (e.g. attitudes, values, norms, behaviour) across individuals.

The most comparable methodological approach in the social identity literature is SIM (Cruwys et al., 2016), which has previously been used as a technique for measuring and mapping social identities at the individual level. That is, each identity map is specific to only one individual. Consequently, these identity maps tell us little, statistically, about the generalizability of relations (i.e. the network edges) between individual identities (i.e. the network nodes). By collecting similar data (i.e. using a bottom‐up, qualitative approach) – but analysing it using network methods – we could quantify the strength of the co‐occurrences *within* individuals, while also aggregating the multiple identities reported *across* individuals. In this sense, we were able to quantify qualitative data. Our approach retains the richness of the data, while extracting identity–identity and identity–behaviour pairings that are statistically meaningful. Consequently, it generates more representative data regarding the identities that are found to occur – and co‐occur – within a given population.

### Theoretical and practical implications

Our data highlight that the broader identity‐by‐identity and identity‐by‐behaviour networks are not significantly correlated overall. Nevertheless, there are a small number of identities associated with performance of the same behaviours – as can be inferred from the networks of *significantly* co‐occurring identities and behaviours. Therefore, while groups may aim to maintain an identity that is positive and distinct at the intergroup level – as described within SIT (Tajfel, 1978; Tajfel & Turner, 1979, 1986) – the degree of behavioural overlap among identities that are shared within‐individuals and closely related (e.g. workplace and psychologist identities engaging in personal development and work‐related behaviour; sports team and athlete identities engaging in exercise and health‐related behaviour; student and age group identities engaging in socializing, partying and drinking behaviour) is nevertheless notable. Traditionally, intergroup relations have been defined in a binary manner – consisting of both ingroups and outgroups. However, multiple ingroups often locate themselves within superordinate identities and develop both hierarchical and lateral relations with other groups (Hornsey & Hogg, 2000) – as captured in models of Social Identity Complexity (Roccas & Brewer, 2002). For example, in organizational settings, multiple subgroups can be found to externally cross cut and assimilate with superordinate categories (Ashforth et al., 2010; Ashforth & Johnson, 2001; Brewer, 1996), and it is unsurprising that groups of this nature may share similar behavioural profiles (e.g. where formal ‘occupational’ identities cross cut with informal ‘work colleague’ identities; see Appendix D). This may be even more evident in samples where subgroup identities are more prevalent (i.e. in American samples, where evangelical, conservative and Republican identities have been found to converge, as have Black, secular, liberal and Democratic identities; Mason, 2015, 2016), and behavioural data are more granular.

These findings may be particularly insightful for those conducting research, and designing interventions, in the context of social identity and behaviour. Data regarding the identities and behaviours found to significantly co‐occur provides a unique resource for those wishing to identify specific groups and behaviours to feature in future social psychological research. To date, population‐level information regarding the multiplicity of our social identities, and their associated behaviours, has been scarce. These findings may also help researchers identify the most appropriate target group for a particular behaviour change intervention. By providing a means of easily identifying the group memberships associated with particularly maladaptive and identity‐infused behaviours – such as alcohol consumption, for example – efforts to change behaviour can be directed towards those who may benefit from them most.

Moreover, in acknowledging the multiplicity of our social identity networks, our findings provide additional insights regarding how best to design these interventions to achieve long‐term behaviour change. Norms‐based interventions, for example, typically target behaviour in relation to a single social identity (e.g. drinking in relation to the student identity; LaBrie et al., 2013; Lewis & Neighbors, 2006; Neighbors et al., 2004). However, these interventions are unlikely to be successful if multiple other cross‐cutting identities are also associated with the performance of this behaviour. Taking drinking behaviour as an example, an intervention that weakens the association between students and drinking is unlikely to reduce an individual's alcohol consumption if the behaviour is also performed by other groups with which they highly identify (e.g. national identities, peer group identities, age‐related identities). Rather, these cross‐cutting identities will continue to reinforce the behaviour, thereby reducing the efficacy of the intervention. This is likely to be most pervasive in more modular networks – that is, where there is a high degree of interconnectivity within sub‐groups. It is likely, however, to be evident even in networks low in modularity, but high in density – such as that of the present research. For example, in our research, we see multiple identities (e.g. student‐teenager‐young adult, family‐partner‐sibling) being linked by the same behaviours irrespective of our low level of modularity. Therefore, network analyses hold important practical implications, and provide a useful resource to identify and target behavioural crossover even in non‐modular networks. Practitioners will benefit from considering the multiplicity of our social identities, the interconnectivity of identity networks and the fact that behaviour may not reliably be the product of a single identity in isolation.

### Modularity in social identity networks

In our sample, neither of the identity‐by‐identity, behaviour‐by‐identity or identity‐by‐behaviour networks demonstrated strong statistical evidence of substructures among groups of identities or behaviours. In the case of our identity‐by‐identity networks in particular, this may be attributed to the broad framing of our research focus. That is, participants were broadly asked to report any groups that they identified with, without specifying any specific levels of abstraction or communities to focus on. While this allowed us to collect data that is representative of the breadth of different social identities that an individual may possess, it may have encouraged participants to report a wider range of more inclusive, superordinate identities. When limited to reporting only the first 10 identities that come to mind, it is also possible that these identities may have been most accessible and salient to participants. Consequently, our networks almost exclusively depicted relations among superordinate identities. Nevertheless, this is not to say that a higher degree of modularity would not exist among identities when sampled in a context that is less abstract, and more specific to particular communities. For example, organizations tend to be hierarchically structured – with work‐team identity often being embedded within the higher order organizational identity (Jetten et al., 2002) – and individuals in the United States are found to develop distinctive identity‐groupings along party lines (Huddy & Bankert, 2017; Mason, 2015, 2016). Specific to university settings, individuals may also identify with particular subgroups relevant to the overarching student identity – such as being an undergraduate/postgraduate student, being a member of a university sports team, attending a drama society and being a housemate. It is, therefore, conceivable that more discreet clusters of identities may emerge when group memberships are sampled in more specific contexts, at lower levels of abstraction.

Furthermore, the low degree of modularity demonstrated in our networks may have been further exacerbated by the way in which the identities and behaviours were collected and re‐categorized during data cleaning. Asking participants to reflect on predetermined group memberships in itself may impact those identifications (Haslam et al., 1999). Hence, rather than asking participants to rate their level of identification with predetermined social identities – and the extent to which these identities are associated with particular behaviours – from the top‐down, identities and corresponding behaviours were qualitatively generated by the participant in a bottom‐up, inductive manner. However, this resulted in an exceptionally large number of identities and behaviours, which would have been challenging to model and visualize in a clear and meaningful way. This issue was particularly evident in the behavioural data, as there was no limit on the number of corresponding behaviours that could be mentioned alongside each identity. Therefore, in instances where more specific and nuanced identities and behaviours *were* reported, the majority of these were re‐categorized under the relevant overarching categories, which typically appeared at a higher frequency. It is possible that this approach to data cleaning may have erased much of the sub‐structuring that would have otherwise been evident (e.g. larger groups of subordinate identities all connected to the same over‐arching superordinate identity). A less restrictive approach to data cleaning may more accurately represent the richness of the data and reveal greater clustering, though the benefit of this should be weighed against the potential costs regarding the clarity of the resultant network models.

### Limitations and future research

The present research has some noteworthy limitations. First, our sample is relatively small, student‐oriented and predominantly UK based. Therefore, our data do not portray a representative population‐level analysis of the co‐occurrences among identities and behaviours. However, having established proof of concept – that network methods are able to take data collected at the level of the individual, and aggregate across individuals to map meaningful relations among social psychological constructs at a wider sample level – future research may wish to implement similar methods on a larger scale to draw stronger conclusions regarding the co‐occurrences found to exist in the general population.

Although our method of data collection represents a strength of the research – in that it did not impose predetermined categories upon participants – the collection of qualitative data regarding an individual's social identities and behaviours resulted in a lengthy data cleaning procedure that was time and resource intensive. As this approach has helped to identify several identities that are found to significantly co‐occur within individuals, future research designs may wish to use this resource to carry these identities forward – adopting a quantitative approach to determining a participant's level of identification with each group membership (e.g. via self‐report measures such as Likert scales, visual analogue scales etc.). If focusing on identities in specific populations or communities, future research may benefit from including predetermined identities of different levels of abstraction (i.e. both subordinate and superordinate identities) to ensure that sub‐groups and clusters of identities and behaviour may be identified if present. In these more specific samples, it may be beneficial to first conduct pilot research – replicating our qualitative approach – to guarantee that all relevant subordinate identities are identified and adequately represented when subsequently collecting quantitative data.

A quantitative approach to the measurement of social identification in future research will also allow researchers to measure dimensions of identity that were not captured here. In the present research, social identification was inferred from group membership; participants freely listed the groups/identities that they belong to. This conceptualization of social identity somewhat aligns with one key aspect of Tajfel's (1978) definition of the construct as “that part of an individual's self‐concept which derives from his [or her] knowledge of his [or her] membership of a social group” (p. 63). However, we acknowledge that a binary conceptualization of social identity (i.e. as either being a group member, or not) is limited and does not align fully with more recent work on the multidimensional nature of social identity (Cameron, 2004; Cameron & Lalonde, 2001; Ellemers et al., 1999; Jackson, 2002; Leach et al., 2008).

One particularly important dimension of social identity to consider here is identity centrality, which is typically operationalized in terms of both the *cognitive accessibility* of group membership (Gurin & Markus, 1989) and the subjective *importance* of the group to self‐definition (Luhtanen & Crocker, 1992). In only capturing the momentary accessibility of our participants' group memberships, our measurement did not reflect that the typical frequency with which a given group comes to mind, and the relative importance of a group, may differ across identities. Future research should assess how central each identity is perceived by the individual to obtain a more comprehensive picture of how identities are interrelated with one another and behaviour. Network analytical methods can then be used to decipher whether central identities co‐occur in the population and whether they are more closely linked via shared behaviour. Given that high centrality among pairs of identities (e.g. woman and scientist; Settles, 2004) is found to buffer against identity interference (e.g. in terms of an individual feeling able to ‘enact’ their identities), higher centrality may be associated with greater behavioural overlap in a network.

Finally, the present research gives some initial indication that identities found to significantly co‐occur at a rate lower than expected by chance may do so because they are incompatible in terms of their associated behaviours. None of the identities found to significantly co‐occur *less often* than expected by chance in identity‐by‐identity networks were found to be significantly associated with the same behaviours *more often* than expected by chance in identity‐by‐behaviour networks. However, the current research design is unable to directly speak to the compatibility of associated identities and behaviours. Identities that are shared are not necessarily compatible, and consequently, mere co‐occurrence of identities is not an appropriate proxy measure of compatibility. Nevertheless, our methodological approach is highly adaptable because it can map several indices regarding the relations between social identities. Future research may wish to use this adaptability to test this supposition (i.e. that identities that are compatible have similar behavioural profiles and identities that are incompatible have distinctive behavioural profiles) directly by asking participants to specify the compatibility of their group memberships relative to one another. Network methods would then be able to map and quantify these relations (e.g. with edge‐weight between nodes in identity‐by‐identity networks representing compatibility/incompatibility) – generating representative data regarding the identities found to be compatible, and incompatible, at a population level.

## CONCLUDING REMARKS

In sum, the current research highlights the utility of network analytic approaches in mapping associations among the social psychological constructs of social identification and group‐based behaviour. The approach is unique and advantageous in that it is able to capture co‐occurrences between identities and behaviours at a wider sample level – allowing us to draw stronger, statistically informed conclusions regarding the overall structure of our identity networks, and the groups that are found to most frequently co‐occur in a given sample. Overall, our network structures were found to be significantly different than expected by chance, but demonstrated little evidence of clustering among different groups of identities or behaviours. Therefore, identity networks appear to be diverse and densely populated; individuals possess multiple different identities and exhibit strong ties both within and between multiple different subgroups. However, this is not to say that this pattern will be consistent across all networks of social identities; future research is required to shed light on the way in which identity networks, and their associated behavioural profiles, may differ structurally across various samples and communities.

## AUTHOR CONTRIBUTIONS

**Emily A. Hughes:** Conceptualization; data curation; investigation; methodology; project administration; resources; writing – original draft; writing – review and editing. **Samuel Ellis:** Data curation; formal analysis; software; visualization; writing – review and editing. **Joanne R. Smith:** Conceptualization; methodology; project administration; resources; supervision; writing – review and editing.

## CONFLICT OF INTEREST STATEMENT

All authors declare no conflict of interest.

### OPEN RESEARCH BADGES

This article has earned Open Data and Open Materials badges. Data and materials are available at https://osf.io/2fe8c/; https://osf.io/mf5hq/.

## Data Availability

The data that support the findings of this study are openly available under the ‘Data & Analysis’ section of the project Open Science Framework (OSF) page at https://osf.io/2fe8c/, Hughes et al. (2018).

## Footnotes

^1^
In this research, we focus on behaviour as an important aspect of identity, and one that may help to explain potential incompatibility among identities. Our measurement of behaviour focuses on capturing behaviour that is seen to be typical of a given group. In effect, our data capture the descriptive norm as part of the content of an identity (Cialdini et al., 1990). This is not to suggest that other aspects of identity content (e.g. attitudes, injunctive norms, values, etc) are not important in understanding identity compatibility. Rather, we believe that the adaptability of our analytic approach provides a means of mapping and disentangling multiple aspects of identity content – and their association with identity compatibility – in future research (see Discussion for elaboration).

^2^
Data were collected between December 2018 and January 2019. Final sample size was determined by the number of participants we were able to recruit within this assigned time frame.

^3^
We also collected data on the behaviours that were atypical for each group membership, but these data are not reported here.

^4^
Forty‐two ‘identity’ names were excluded from all analyses due to either being (a) adjectives (e.g. small, minimalistic, hard‐working) rather than group memberships, or (b) too broad and/or not tied to a particular group per se. A further 51 were excluded from behaviour‐by‐identity and identity‐by‐behaviour analyses due to these identities not possessing any corresponding behaviours. A spreadsheet outlining the excluded identities can be found under the materials section of the project OSF page. The full data‐cleaning procedure can be found in Appendix A.

^5^
Modularity was calculated for both unfiltered and filtered networks for comparison.

^6^
Only filtered networks resulting from our re‐analysis are presented for ease of interpretation, given that data filtering reduces the total node count.

^7^
As identities are unequally sampled, other network noise reduction methodologies – such as ‘backboning’ (see Serrano et al., 2009) – are not appropriate. It is possible that these methods could be adapted to uncover structure in equally sampled networks and give insights into hidden structures in social identity networks. However, conceptually creating networks ‘significant’ relations (i.e. of identities and behaviours co‐occurring significantly more, and less, frequently than expected by chance) – as conducted here – has a similar effect of revealing structure beneath the noise of the larger network.

^8^
The structure of the networks (i.e. in terms of density and modularity) remained constant when identities were re‐coded to reflect more abstract, higher order categories. Network structure is not dependent on the categories used in our original data‐cleaning procedure. Only our primary analyses are presented in‐text. Network figures and summary statistics pertaining to the re‐analysis of data using higher order identity categories can be found in Appendix D, and should be interpreted similarly.

^9^
We follow Newman (2004) who suggests that modularity >.30 should be considered as evidence of meaningful sub‐structuring in the network.

## APPENDIX A Data‐Cleaning Procedure

As participants were asked to report their group memberships and corresponding behaviours qualitatively, a large number of identities and behaviours were recorded. However, this presented a challenge for data analysis for the following reasons: (a) participants would often describe the same identity or behaviour in different words, (b) participants sometimes entered descriptors that were not identities or behaviours per se, (c) the data analysis software is case sensitive and would, therefore, recognize capitalized and uncapitalized versions of the same word as being different (e.g. ‘Drinking’ and ‘drinking’ would be recognized as two separate behaviours) – which greatly inflates the number of reported identities and behaviours – and (d) a large number of identities and behaviours would be difficult to model graphically and draw meaningful conclusions from. Therefore, identity and behaviour data were re‐categorized and standardized to reduce the total number of categories and to clearly represent only meaningful identities and behaviours. All data cleaning was carried out by a single coder, and checked by the first author.

The primary aim was to re‐categorize *subordinate* identities and behaviours (i.e. those originally generated by participants in the raw data set) into *superordinate* equivalents (i.e. overarching category titles that are representative of the underlying subordinate entries). In order to re‐categorize identities, all identities were listed as written by participants, and commonalities were identified among them. Thirty entries deemed not to be identities per se were highlighted at this stage, and deleted from the final data set. Forty‐two entries could be interpreted as identities, but were ambiguous in that they either: (a) better‐represented adjectives (e.g. small, minimalistic, hard‐working) rather than group memberships, or (b) were too broad and/or not tied to a particular group, were coded red in the final data set. These identities were not deleted from the final data set but were excluded from analyses. Superordinate category titles were then generated by the coder, and the subordinates comprising each new superordinate were listed for reference. Finally, identity entries were re‐coded in the final data set. In a second round of data cleaning, several of the superordinate identities were collapsed to further merge and reduce the number of identities to ensure clarity of graphical representation. These changes are not reflected in the final data set; identities were instead re‐coded during data analysis. The spreadsheets used to record the initial re‐categorization of identity data (i.e. the first round of data cleaning), and subsequent collapsing and renaming of identities (i.e. the second round of data cleaning), can be found under the materials section of the project OSF page.

In order to re‐categorize behaviours, all behaviour subordinates were tabulated next to their corresponding identities as written by participants. Subordinate behaviours were then assessed for prevalence (i.e. frequency of appearance and the extent to which they are consensually performed among group members) and commonality in phrasing; the former was denoted next to where the behaviour was listed. Entries deemed to not be behaviours per se were highlighted in red within the re‐categorization table, and deleted from the final data set. Superordinate category titles were then generated by the coder; it was advised they cap the number of superordinate behaviours per identity to 10 for simplicity. In a final column, the subordinates comprising each new superordinate were listed for reference. Finally, behaviour entries were re‐coded in the final data set. The table used to document the re‐categorization of behavioural data can be found under the materials section of the project OSF page.

## APPENDIX C Data Cleaning: Identity Re‐Coding Procedure

After our initial two rounds of identity data cleaning (see Appendix A), the data were collapsed further to explore the broader generalizability of our observed network structure across individuals and determine whether this structure depends on the categories used in our original data‐cleaning procedure.
^10^
We thank an anonymous reviewer for this suggestion.

Our initial re‐categorization procedure coded identities at the highest level of abstraction specified by participants. For example, the various nationalities mentioned by participants (e.g., British, American, Canadian) were retained as individual nodes within the network, as opposed to being collapsed under the higher order category title of ‘nationality’. At a more abstract level, however, these participants all identified with their nationality. We took this approach to avoid imposing category titles onto participants and to retain some of the richness and granularity we obtained through the use of open‐text boxes during data collection. However, we acknowledge that the density of the identity networks may make it more difficult to understand the identities that are associated on a meta‐level. It also may impede our ability to identify meaningful subgroups (i.e. modularity) in the data, given that some lower order identities are mutually exclusive, and therefore unlikely to be associated with one another. For example, if an individual identifies as ‘working‐class’, then they are unlikely to also identify as ‘upper‐class’.
^11^
Assuming class‐related categories to be largely mutually exclusive, which we acknowledge may not *always* be the case. Where this is true, one would not expect to see associations, or clustering, among class‐related identity nodes. It would, nevertheless, be meaningful to understand the other identities that are associated with ‘class status’ more broadly, irrespective of the class level that is identified with. We re‐coded the identity categories to reflect the overarching categorical themes within the data (i.e., adopting a more abstract, higher‐order level of identity categorization). This allowed us to compare the different categorization approaches, and explore whether different levels of categorization abstraction would reveal different underlying network structures.

Lower order identities were re‐coded under the corresponding higher order categories where they were agreed to have a high likelihood of being mutually exclusive, and therefore not associated with one another. This re‐coding was carried out by the first author, and checked by both co‐authors. Re‐coding resulted in the inclusion of the following higher order categories: gender, race and ethnicity, nationality, British region, British city, student, political orientation, class, occupation, work colleague, sexual orientation, hair colour, school, employment status, educated, age group, religious belief and relationship status. The spreadsheet used to record the re‐coding (i.e. collapsing and renaming) of identities to reflect the more abstract, higher order categories that they represent can be found under the materials section of the project OSF page.

As noted in the main text, re‐coding did not alter the substantive pattern of results regarding the structure of our networks (i.e. in terms of density or modularity; see Appendix D).

## APPENDIX D Filtered Network Figures: Identity Re‐Coding Re‐Analysis

### Identity‐by‐Identity Network

N nodes = 49.

Density = 0.53.

Modularity = 0.16.

### Behaviour‐by‐Identity Network

N nodes = 28.

Modularity = 0.10.

Density = 0.74.

### Identity‐by‐Behaviour Network

N nodes = 40.

Modularity = 0.10.

Density = 0.38.
